# Socioeconomic inequalities in healthcare utilization among older adults in Indonesia: social network mediation and dynamics before and after Universal Health Coverage

**DOI:** 10.1186/s12913-026-16046-0

**Published:** 2026-11-11

**Authors:** Julia Schröders, Miguel San Sebastian, Fatwa Sari Tetra Dewi

**Affiliations:** 1 Department of Epidemiology and Global Health, Umeå University, Umeå, 901 87 Sweden; 2 Department of Health Behaviour, Environment, and Social Medicine, Faculty of Medicine, Public Health and Nursing, Universitas Gadjah Mada, Yogyakarta, Indonesia

**Keywords:** Social networks, Healthcare utilization, Socioeconomic inequalities, Social network dynamics, Universal health coverage, Ageing, Indonesia, Mediation analysis

## Abstract

**Background:**

Socioeconomic inequalities in healthcare utilization (HCU) remain widespread in many low- and middle-income countries, even following major health system reforms. Social networks may influence individuals’ ability to access healthcare by providing information, resources, and support, yet their role in shaping socioeconomic inequalities in HCU remains poorly understood. This study examined whether social networks and their dynamics over time mediate the relationship between socioeconomic position (SEP) and HCU among older adults in Indonesia across periods before and during the implementation of Universal Health Coverage.

**Methods:**

Data were drawn from the fourth (2007–2008) and fifth (2014–2015) waves of the Indonesian Family Life Survey, including 7,964 and 7,986 adults aged 50 and older, respectively. HCU outcomes included outpatient service use and participation in the posyandu lansia programme during the previous four weeks. SEP was modelled as a latent construct based on education, household expenditure, and wealth indicators. Social networks were conceptualized across four ecological levels (individual, family, community, and societal), and social network dynamics were assessed using a panel sample of 4,411 individuals participating in both waves. Causal mediation analysis was applied to estimate the proportion of the SEP–HCU relationship mediated by social network characteristics.

**Results:**

HCU was higher in IFLS-5 than in IFLS-4, with outpatient service use rising from 16.9% to 24.6% and posyandu participation from 2.5% to 6.7%. Socioeconomic inequalities persisted, with higher SEP groups more likely to use outpatient services. Social network dynamics were common, with 36% of respondents experiencing changes over time, and were socially patterned, with lower SEP groups more likely to show increasing network trajectories. Loneliness and marital status mediated up to 7.3% of the association between SEP and outpatient service use, while community-level social networks mediated up to 21.7% of the relationship with posyandu participation.

**Conclusions:**

Social networks contribute to explaining socioeconomic inequalities in HCU among older adults in Indonesia. The findings highlight the importance of both structural health system reforms and informal social support systems in shaping HCU. Strengthening community networks and addressing social isolation may complement improvements in formal healthcare coverage and help promote more equitable HCU in ageing populations.

**Supplementary Information:**

The online version contains supplementary material available at https://doi.org/10.1186/s12913-026-16046-0.

## Background

Humans are social animals and, based on this premise, the scientific community has produced extensive evidence over the past five decades demonstrating the strong relationship between social networks and health [1–6]. Social relationships influence health through multiple mechanisms, including the diffusion of health behaviours, provision of emotional and instrumental support, and facilitation of access to resources and services. Particularly in Europe [7–9] and the United States [10–12], longitudinal studies have highlighted the importance of social networks for health outcomes among ageing populations. Although evidence from low- and middle-income countries (LMICs) remains comparatively limited, an increasing number of studies are beginning to explore the role of social networks in shaping health outcomes in these contexts [13, 14]. Our previous work in Indonesia, for example, has demonstrated the importance of social network diversity for a wide range of health outcomes among older adults [15].Due to growing insights into the associations between social networks and physical health outcomes [16, 17], mental health conditions [18, 19], health behaviours [20, 21], and mortality [22], there has been increasing interest in understanding whether social networks also influence patterns of healthcare utilization (HCU).

Previous studies examining HCU have predominantly focused on individual health behaviours and have therefore often been informed by theoretical frameworks such as Fishbein’s Theory of Reasoned Action [23] or Andersen’s behavioural model of healthcare utilization [24]. However, these models give limited attention to the relational and social contexts within which healthcare decisions are made. From a social network perspective, there are strong theoretical reasons to expect that social relationships influence how individuals navigate healthcare systems and make decisions about seeking care. Two conceptual frameworks are particularly relevant in this regard: Berkman’s conceptual model of social networks and health [1] and the Network Episode Model (NEM) [25]. While the Berkman model emphasizes pathways through which social networks shape health-related behaviours, attitudes, and resource flows, the NEM highlights the interpersonal processes within social networks that influence decision-making during illness episodes. Together, these perspectives extend traditional health behaviour models by emphasizing the broader socio-structural context in which healthcare decisions occur.

The relationship between social networks and HCU has been conceptualized differently across settings. In many high-income countries, socially isolated or lonely older adults are often described as placing increased demands on health and social care services, and interventions have therefore sought to improve the quantity and quality of social connections to reduce inappropriate service use [26–28]. In contrast, in many LMIC settings social networks are often viewed as essential safety nets that facilitate access to healthcare in contexts where formal support structures remain limited [14]. In such contexts, family members, neighbours, and community networks may play a critical role in recognizing health needs, mobilizing resources, and facilitating pathways into care.

When examining the link between social networks and HCU in LMIC settings such as Indonesia, it is also crucial to consider socioeconomic inequalities. Large socioeconomic disparities in healthcare utilization have been documented in many LMICs [29, 30], including Indonesia [31, 32]. Individuals with higher socioeconomic position (SEP) are often more likely to access healthcare services due to differences in financial resources, health literacy, and social capital. In response to these inequalities, Indonesia introduced a national Universal Health Coverage (UHC) programme in 2014 through the implementation of the National Health Insurance scheme (*Jaminan Kesehatan Nasional*, JKN), aiming to reduce financial barriers to healthcare access, particularly for vulnerable populations including older adults and individuals with chronic conditions [33–35]. In addition to formal healthcare services, Indonesia has a long-standing system of community-based integrated health posts (*posyandu*). The *posyandu* platform has operated since the 1980s, initially focusing primarily on maternal and child health, nutrition, immunization, and family planning, and was subsequently extended to older populations. *Posyandu lansia*, specifically targeting older adults, was established prior to the introduction of JKN and was already operational during the IFLS-4 period [36, 37]. As an established community-based delivery platform, *posyandu* has played an important role in extending primary healthcare outreach to village and rural populations, including groups facing barriers to formal healthcare services. The programme provides community-based preventive and promotive services for older adults, including health monitoring, non-communicable disease screening, health education, and referral where needed. Its continued operation alongside the expansion of JKN therefore provides an important complementary indicator of healthcare utilization across the two study periods. Its continued operation alongside the expansion of JKN therefore provides an important complementary indicator of healthcare utilization across the two study periods. These health system reforms provide an important opportunity to examine how social determinants—including social networks—interact with structural health system changes to influence patterns of healthcare utilization.

There is also a considerable body of research documenting the socioeconomic patterning of social networks [38, 39], although empirical findings remain mixed [40, 41]. In Indonesia, however, health-related social network research remains limited [42]. Early anthropological observations described strong norms of resource sharing among poorer communities, sometimes referred to as “shared poverty” [43, 44]. More recent research suggests that processes of economic transformation, migration, and demographic change may be altering these traditional patterns of social support, potentially reshaping social networks and their capacity to provide care and assistance [45, 46].

Beyond the structural characteristics of social networks, their dynamics over time may also play an important role in shaping health behaviours and healthcare utilization. Social network changes frequently occur in response to normative and non-normative life events across the life course [47]. While earlier theories suggested that social networks shrink in old age due to disengagement [48], more recent perspectives emphasize processes such as socioemotional selectivity, in which older adults prioritize emotionally meaningful relationships [49]. At the same time, ageing is often accompanied by life transitions such as retirement, widowhood, or declining health that may reshape both social networks and socioeconomic circumstances.

To the best of our knowledge, no study in Indonesia has simultaneously examined the interplay between socioeconomic position, social networks, and healthcare utilization while also considering social network dynamics over time. Addressing this gap is important for understanding how social determinants influence healthcare access in ageing populations within rapidly changing health systems. We conceptualized social networks using a multilevel systems approach encompassing individual, family, community, and societal dimensions [50]. Therefore, this study aimed to examine the role of social networks in the relationship between socioeconomic position (SEP) and healthcare utilization (HCU) among older adults in Indonesia across two periods before and after the introduction of Universal Health Coverage. Specifically, we addressed three research questions: [1] How did socioeconomic differences in outpatient healthcare utilization and posyandu lansia participation differ between the pre-UHC period (IFLS-4, 2007/08) and the period following the introduction of UHC (IFLS-5, 2014/15) [2]? To what extent did social network characteristics at the individual, family, community, and societal levels mediate the association between SEP and HCU in each period? and [3] How were changes in social networks between IFLS-4 and IFLS-5 patterned by SEP, age, and sex/gender, and to what extent did these network dynamics mediate the association between SEP and HCU in the later period?

## Methods

### Data source

This study used data from the fourth and fifth waves of the Indonesian Family Life Survey (IFLS), fielded in 2007–2008 (IFLS-4) and 2014–2015 (IFLS-5) [51, 52]. The IFLS is an ongoing longitudinal survey of individuals, households, and communities and is representative of approximately 83% of Indonesia’s non-institutionalized population living in 13 provinces [53]. The survey was designed to capture the country’s substantial socioeconomic, geographic, and cultural diversity. The IFLS employs a multistage stratified sampling design based on province and rural–urban classifications. The sampling frame was derived from the enumeration area (EA) list of the National Socioeconomic Survey (SUSENAS). Rural areas were slightly oversampled, with 30 households (instead of 20) selected per EA to ensure adequate representation of rural populations. Both waves of the survey achieved high response rates. Participation rates were approximately 92% in both IFLS-4 and IFLS-5, and the re-contact rate between waves was 91%, indicating relatively low attrition [51, 54]. The IFLS allows both longitudinal panel analyses and repeated cross-sectional analyses. All IFLS data are publicly available through RAND (www.rand.org/labor/FLS/IFLS). Ethical approval for IFLS-4 and IFLS-5 was obtained from the Institutional Review Boards of the RAND Corporation and Universitas Gadjah Mada, in collaboration with SurveyMETER (ethical approval no. s0064-06-01-CR01). The surveys were conducted in accordance with the principles of the Declaration of Helsinki, and written informed consent was obtained from all participants prior to data collection.

### Measures

#### Outcome variables: healthcare utilization

Healthcare utilization (HCU) was assessed in both IFLS waves. Two outcome variables were included: (i) outpatient healthcare utilization during the previous four weeks, and (ii) utilization of integrated health services for older adults (*posyandu lansia*) during the previous four weeks. Outpatient care included visits to primary and secondary healthcare facilities, such as public health centres, medical centres, polyclinics, public and private hospitals, as well as consultations with private physicians, nurses, and traditional practitioners. The *posyandu lansia* programme provides community-based preventive and promotive services for older adults, particularly targeting non-communicable disease (NCD) screening and health promotion. Both outcomes were dichotomized to indicate whether respondents had utilized the service during the previous four weeks. Healthcare utilization was treated as an indicator of service use rather than as an inherently desirable outcome, as the analyses did not account for individual healthcare need or intention to seek care.

#### Independent variable: socioeconomic position (SEP)

Socioeconomic position (SEP) was modelled as a continuous latent construct based on three indicators: (i) educational attainment; (ii) household per capita expenditure; and (iii) household wealth. The internal consistency of the SEP construct was acceptable, with Cronbach’s alpha values of 0.70 (2007–2008) and 0.72 (2014–2015). Educational attainment was categorized into four groups: no schooling; elementary education; secondary education; and university education. Household per capita expenditure (PCE) was calculated by IFLS based on monthly household consumption expenditures, including food and non-food consumption, durable goods, services, housing, and education expenditures [55]. Total expenditures were divided by household size and categorized into quartiles, with the lowest quartile representing the poorest 25% of respondents. Household wealth was measured using an asset-based wealth index constructed via principal component analysis (PCA) [56]. The index included household amenities (e.g., water source, sanitation, cooking facilities, ownership of refrigerator, television, and car) as well as housing characteristics (e.g., building materials and housing condition). Wealth scores were divided into quintiles, with the lowest quintile representing the poorest households. Following recommendations by Krieger [57] and Galobardes [58], these indicators were combined to form a composite SEP measure capturing both long-term and short-term dimensions of socioeconomic stratification.

#### Mediators: social networks and social network dynamics

Following a multilevel systems approach to social relationships [50], social networks were conceptualized across four ecological levels: (i) individual level; (ii) family level; (iii) community level; and (iv) societal level.

##### Individual level

At the individual level, we included a subjective measure of loneliness, assessed through respondents’ reports of feeling lonely during the previous week. Response categories ranged from no feelings of loneliness to feeling lonely most days of the week. For analytical purposes, responses were dichotomized into “no loneliness” versus “loneliness.” Although loneliness reflects subjective perceptions rather than structural network characteristics, it represents an important psychosocial correlate of social connectedness and has been widely linked to health behaviours and healthcare use [17, 22, 26–28].

##### Family level

Two family-level indicators were included: Marital status (spousal dyad) and contact frequency with adult children. Marital status distinguished between respondents: in a partnership (cohabiting or married, including civil, religious, or customary marriages) vs. single (never married or separated/divorced). Widowhood was treated as a separate category because it represents a major life transition with important implications for social support and network structure. Contact frequency with non-co-resident adult biological children was measured based on both physical and telephone contact during the previous year. Contact frequency was categorized as: high (daily to once per week); medium (at least once per month); or low (once per year or never).

##### Community level

At the community level, we assessed participation in community-based social activities, which represent opportunities for social interaction and social support formation. Three types of participation were included: participation in community programmes (e.g., neighbourhood activities, village meetings, voluntary labour); participation in the *arisan* (rotating savings and credit associations) [59]; and participation in religious activities (e.g., prayer groups). These measures were combined into a community social participation index using PCA, and the resulting scores were categorized into tertiles representing high, medium, and low participation levels.

##### Societal level

At the societal level, we included an aggregated measure of social trust, derived from IFLS questions assessing perceptions of trust and reciprocity within communities. Respondents rated statements regarding: willingness of neighbours to help one another; expectations of exploitation; willingness to ask neighbours for assistance; perceptions of neighbourhood safety; trust toward members of other ethnic groups. Responses were measured on a four-point Likert scale. Individual responses were aggregated to the province level to generate a contextual indicator of social trust representing broader social environments in which individuals are embedded.

##### Social network index and network dynamics

To capture overall network characteristics, we constructed a social network index (SNI) by combining all social network variables. Each component contributed equally to the social network index; no differential weights were assigned to individual network indicators. Separate indices were calculated for IFLS-4 and IFLS-5. Based on data-driven median splits, the indices were categorized into “high” and “low” social network index scores. Social network dynamics were assessed using the panel sample by comparing respondents’ SNI values across the two waves. Changes were calculated by subtracting SNI scores in IFLS-5 from IFLS-4. Network dynamics were categorized as: increasing (score ≥ 1); decreasing (score < 0); or constant (score = 0). Respondents with constant scores were further classified into: constant low networks or constant high networks.

#### Moderators

Two potential effect modifiers were considered: sex/gender (men, women) and age (50–57 years and ≥ 57 years). The age threshold of 57 years was selected because it corresponds to the average retirement age in Indonesia around 2014.

#### Analytical strategy

Analyses comprised two distinct components. First, IFLS-4 (*n* = 7,964) and IFLS-5 (*n* = 7,986) were analysed separately as repeated cross-sectional samples to examine socioeconomic differences in healthcare utilization and the mediating role of social network characteristics at each survey wave; the two waves were not pooled in these analyses. Second, the longitudinal panel sample of 4,411 respondents who participated in both waves was used to derive a person-level measure of social network dynamics based on changes in the social network index between IFLS-4 and IFLS-5. The longitudinal component therefore captured change in social network characteristics rather than repeated healthcare-utilization outcomes. Following previous research [60], IFLS-4 and IFLS-5 were treated as representing pre-UHC and post-UHC periods, respectively. Indonesia introduced its national health insurance scheme (JKN) in January 2014, approximately nine months before IFLS-5 fieldwork began. SEP-related differences in healthcare utilization and social network dynamics were examined using chi-square tests. Associations were further assessed using logistic regression models adjusted for age and sex/gender, reporting odds ratios (ORs) with 95% confidence intervals (95% CI). Statistical significance was defined as *p* < 0.05. Province fixed effects were not included because regional contextual variation formed part of the hypothesized pathways of interest; in particular, the societal-level social trust measure was defined at the province level. Associations between SEP and healthcare utilization were assessed using logistic regression models adjusted for age and sex/gender, reporting odds ratios (ORs) with 95% confidence intervals (95% CI). For individual *i*, the adjusted model was specified as: p < 0.05. Province fixed effectswere not included because regional contextual variation formed part of the hypothesized pathways ofinterest; in particular, the societal-level social trust measure was defined at the province level.

Associations between SEP and healthcare utilization were assessed using logistic regression modelsadjusted for age and sex/gender, reporting odds ratios (ORs) with 95% confidence intervals (95% CI). Forindividual i, the adjusted model was specified as:

$$\operatorname{logit}\{P(Y_i=1)\}= \beta_0 + \beta_1 SEP_i + \beta_2 Age_i + \beta_3 Sex_i$$

where Yi denotes the respective binary healthcare-utilization outcome. Statistical significance was definedas p<0.05.

The primary analytical approach was causal mediation analysis, which allows decomposition of the association between an exposure and an outcome into direct and indirect pathways [61]. We employed a counterfactual-based causal mediation framework, following approaches developed by Imai and colleagues [62–64]. For each social network mediator *M*_*i*_, the mediation analysis consisted of a mediator model and an outcome model. The mediator model was specified as:

$$g\{E(M_i)\} = \alpha_0 + \alpha_1 SEP_i + \alpha_2 Age_i + \alpha_3 Sex_i$$

and the healthcare-utilization outcome model as:

$$\operatorname{logit}\{P(Y_i=1)\}= \gamma_0 + \gamma_1 SEP_i + \gamma_2 M_i+ \gamma_3 Age_i + \gamma_4 Sex_i$$

where *g(*·*)* denotes the link function appropriate to the scale of the mediator, represents the respective social network mediator, and represents the binary healthcare-utilization outcome.

**Fig. 1:**
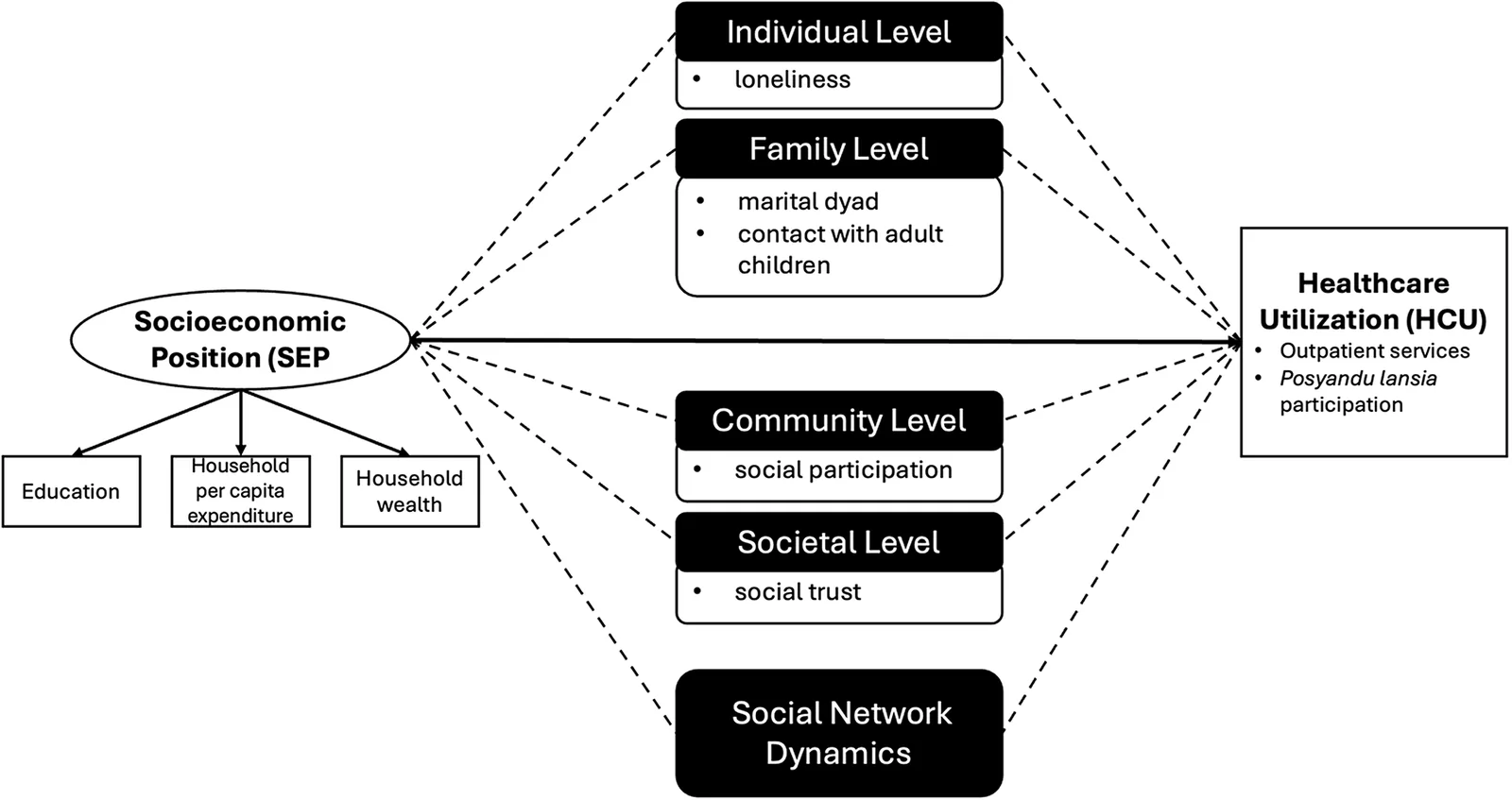
Conceptual framework of the mediation analysis examining the relationship between socioeconomic position and healthcare utilization among older adults in Indonesia. Social networks were conceptualized across four ecological levels: individual (loneliness), family (marital status and contact with children), community (community social participation), and societal (social trust). Social network dynamics represent changes in the social network index between IFLS-4 (2007–2008) and IFLS-5 (2014–2015)

In this framework the average causal mediated effect (ACME) represents the indirect effect through the mediator; and the average direct effect (ADE) represents the effect of the exposure independent of the mediator. Because mediation models with binary outcomes produce standardized estimates, transforming these estimates into odds ratios is not always meaningful. Therefore, we report the proportion of the total effect mediated (PM) along with 95% confidence intervals. The PM is calculated as: PM = indirect effect / total effect; and represents the proportion of the total effect operating through the mediator. A graphical representation of the mediation framework is shown in Fig. 1.

Identification of the natural direct and indirect effects assumes no unmeasured confounding of the mediator–outcome relationship, conditional on SEP and the measured covariates. Because this assumption cannot be empirically verified, we conducted sensitivity analyses for the principal mediation models with binary mediators and binary healthcare-utilization outcomes using mediational E-values [65]. Mediational E-values quantify the minimum strength of association, on the risk-ratio scale, that an unmeasured mediator–outcome confounder would need to have with both the mediator and outcome, conditional on the measured covariates, to fully explain away the estimated indirect effect. Natural indirect effects for these sensitivity analyses were estimated on the risk-ratio scale using parametric g-computation with adjustment for age and sex/gender, and 95% confidence intervals were obtained using 1,000 bootstrap repetitions. Sensitivity analyses were restricted to binary mediators, as the approach does not directly accommodate the four-category social network dynamics measure. All mediation analyses were conducted using the *medeff* command in Stata/SE version 14.2 (StataCorp, College Station, TX) [66].

## Results

Analyses were based on two repeated cross-sectional samples and one longitudinal panel sample. The IFLS-4 sample included 7,964 individuals aged 50 years and older, the IFLS-5 sample included 7,986 individuals, and the panel dataset comprised 4,411 respondents who participated in both survey waves. Descriptive characteristics of the three study samples are presented in Table 1.

**Table 1:** Descriptive characteristics of the IFLS-4, IFLS-5, and longitudinal panel samples

Characteristic	IFLS-4, 2007/08 (*n* = 7,964)	IFLS-5, 2014/15 (*n* = 7,986)	Panel sample (*n* = 4,411)
Age, years, mean (SD)	62.3 (9.8)	61.8 (9.7)	66.9 (8.3)
Sex, n (%)			
Men	3,654 (46%)	3,675 (46%)	2,029 (46%)
Women	4,310 (54%)	4,311 (54%)	2,382 (54%)
Urban residence, n (%)	3,909 (49%)	3,542 (44%)	2,329 (53%)
Rural residence, n (%)	4,055 (51%)	4,444 (56%)	2,082 (47%)

### Healthcare utilization before and after UHC

**Fig. 2:**
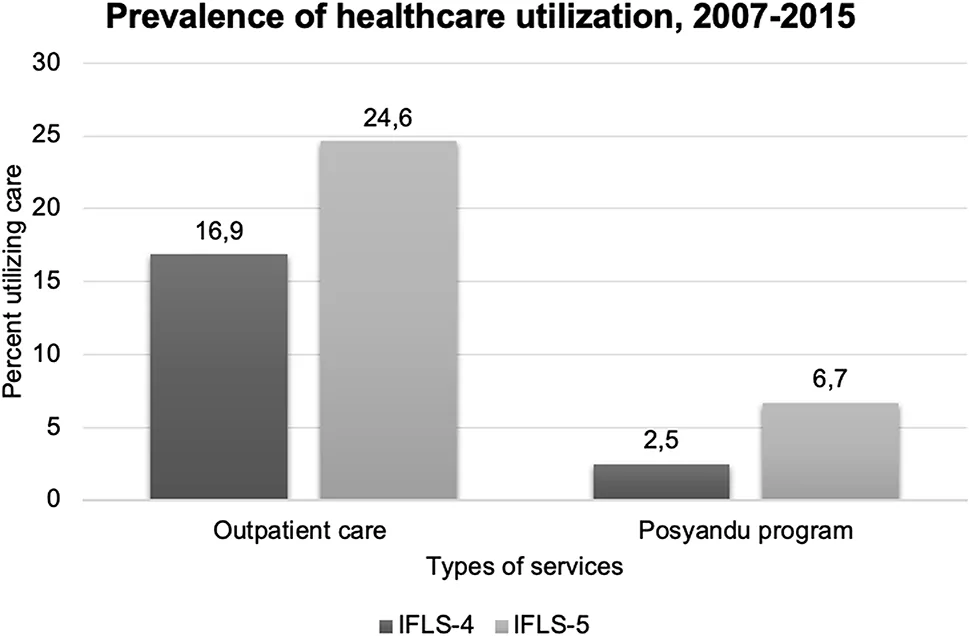
Unadjusted prevalence of outpatient healthcare utilization and posyandu lansia participation in IFLS-4 (2007–2008) and IFLS-5 (2014–2015)

Figure 2 presents the prevalence of healthcare utilization in 2007/08 and 2014/15. The proportion of respondents utilizing outpatient services increased significantly from 16.9% to 24.6% (*p* < 0.001) between the two survey waves. Utilization of the *posyandu lansia* programme also increased, although at lower levels, rising from 2.5% to 6.7% (*p* < 0.001).

Clear socioeconomic gradients were observed for outpatient healthcare utilization. Prior to UHC implementation, outpatient service utilization increased from 11.7% among individuals in the lowest SEP quintile to 20.5% among those in the highest quintile. After UHC implementation, the corresponding proportions increased to 17.6% and 30.6%, respectively (*p* < 0.001). Logistic regression analyses confirmed these patterns. Individuals in the highest SEP quintile had significantly higher odds of utilizing outpatient services both before UHC implementation (OR = 1.79, *p* = 0.015) and after UHC implementation (OR = 1.93, *p* = 0.003) compared with those in lower SEP groups.

In contrast, utilization of the *posyandu lansia* programme showed an inverse socioeconomic pattern, with higher participation among individuals with lower SEP. Utilization rates were 3.3% and 8.4% among the lowest SEP group before and after UHC, compared with 0.7% and 4.9% among the highest SEP group (*p* < 0.001). Regression analyses confirmed these patterns, with lower SEP groups showing higher odds of participating in *posyandu* services.

### Social network characteristics and dynamics

**Fig. 3:**
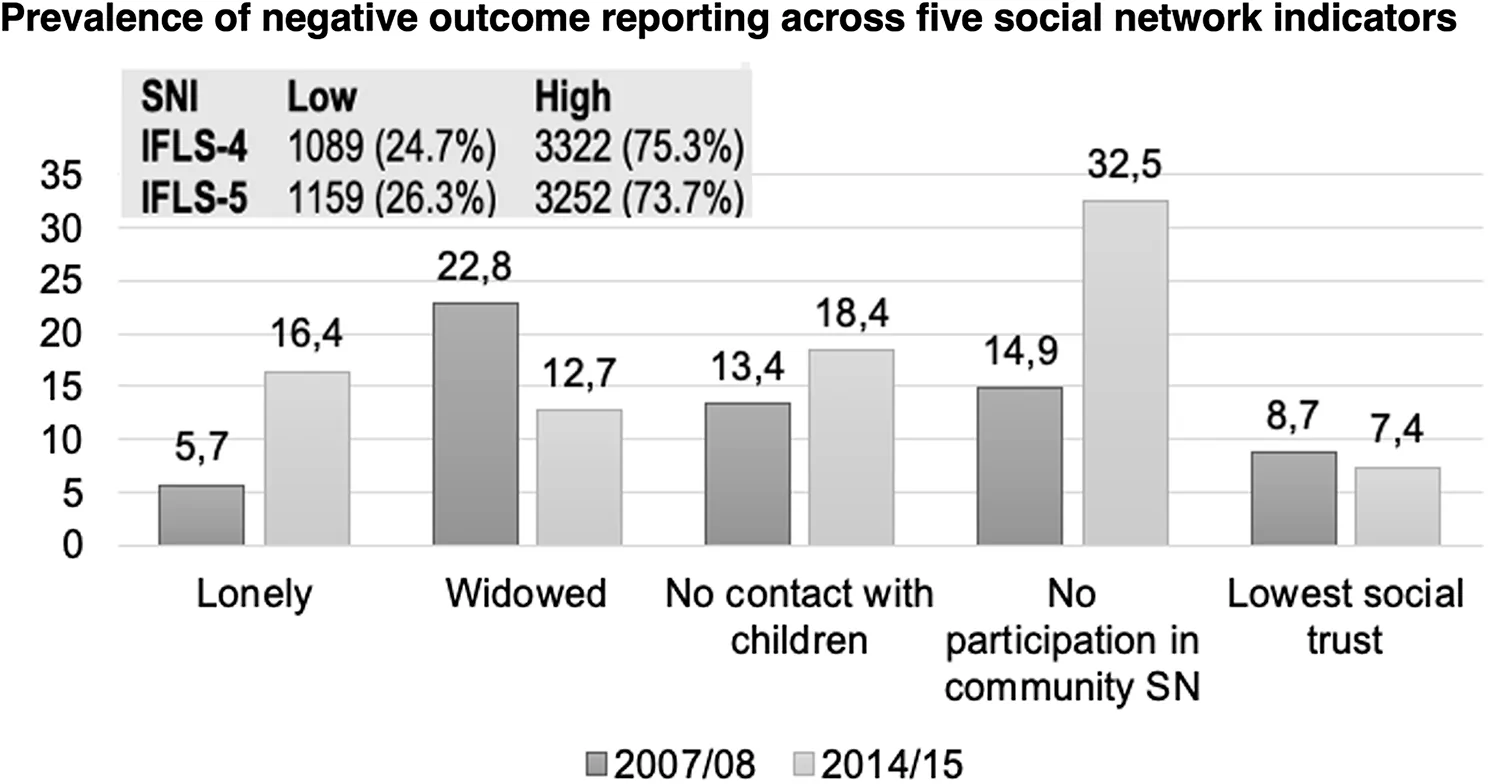
Unadjusted prevalence of adverse social network characteristics in IFLS-4 (2007–2008) and IFLS-5 (2014–2015)

Figure 3 shows the prevalence of negative outcomes across the five social network indicators. Overall, the proportion of respondents classified as having low social network index (SNI) scores increased slightly from 24.7% in IFLS-4 to 26.3% in IFLS-5 (*p* < 0.003). Among the panel sample, 36% of respondents (*n* = 1,602) experienced changes in their social networks over the eight-year period. These changes were categorized as decreasing networks (*n* = 836; 19%) and increasing networks (*n* = 766; 17%). Table 2 presents social network dynamics across socioeconomic groups. Respondents in the highest SEP quintile showed more stable social network patterns, with 66% remaining in the constant high SNI group, compared with 51% among those in the lowest SEP quintile. Conversely, increasing social network trends were more common among lower SEP groups, ranging from 11% in the highest SEP quintile to 22% in the lowest quintile. Only 7% of the sample (*n* = 323) remained in the constant low SNI group, with relatively small differences across SEP groups.

**Table 2:** Unadjusted distribution of social network dynamics across baseline socioeconomic position quintiles in the longitudinal panel sample (n = 4,411)

Social Network Dynamics					
SEP	Constant low	Decreasing	Increasing	Constant high	Total
Q1	68 (8)	172 (19)	201 (22)	463 (51)	904
Q2	60 (7)	173 (20)	156 (18)	495 (56)	884
Q3	66 (7)	171 (19)	161 (18)	488 (55)	886
Q4	76 (9)	163 (19)	155 (18)	464 (54)	858
Q5	53 (6)	157 (18)	93 (11)	576 (66)	869
Total	323 (7)	836 (19)	766 (17)	2486 (57)	4411 (100)

Q1 represents the lowest socioeconomic position and Q5 the highest. Values are presented as n (%)

### Sex and age differences in social network dynamics

**Fig. 4:**
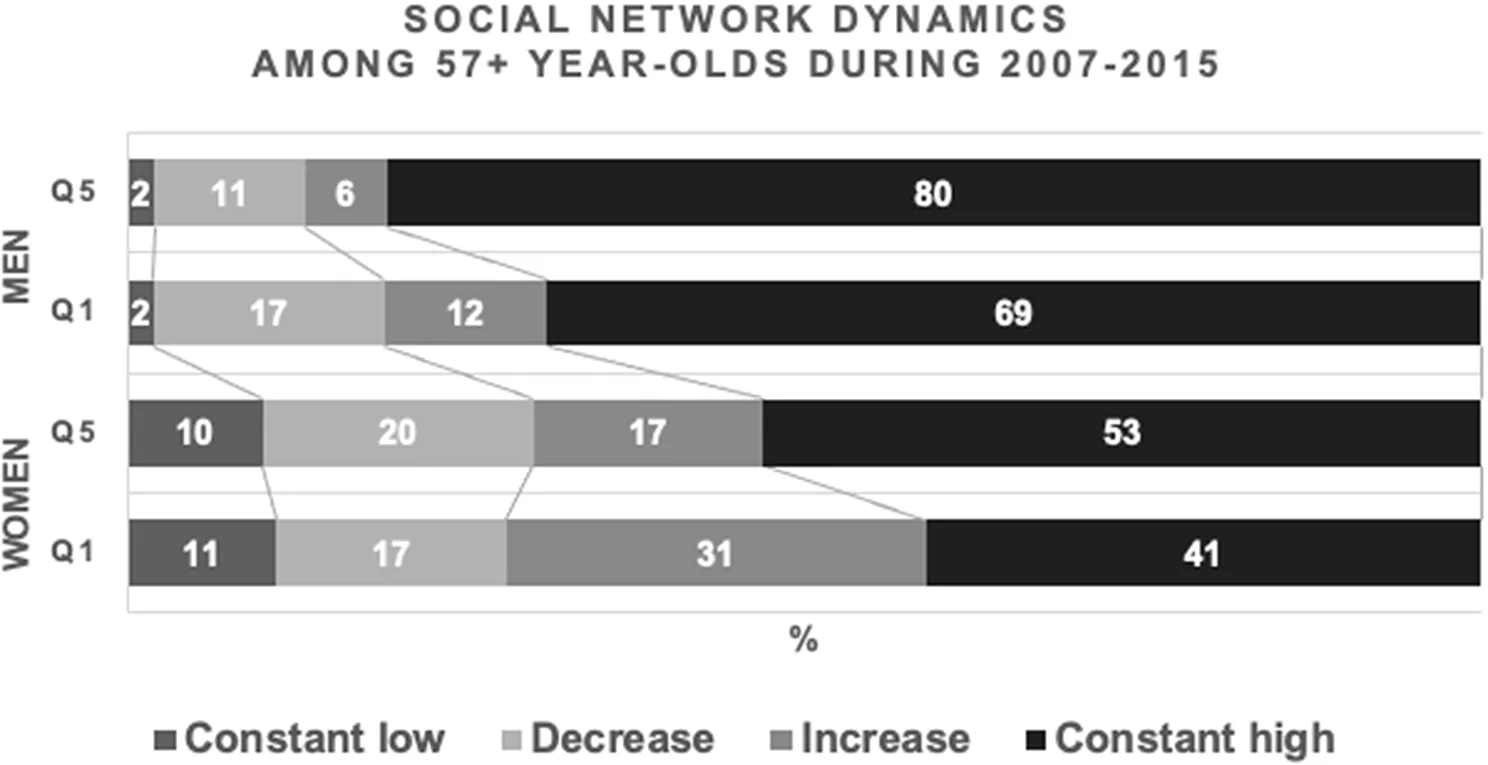
Unadjusted distribution of social network dynamics among adults aged ≥ 57 years in the lowest (Q1) and highest (Q5) socioeconomic position quintiles, stratified by sex/gender

Further stratified analyses (Fig. 4) showed that social network dynamics varied across socioeconomic groups by sex/gender and age. Significant sex/gender differences in network dynamics were observed within SEP quintiles (women: *p* < 0.001; men: *p* = 0.016). When age was additionally considered using a threshold of 57 years, SEP-patterned network dynamics remained statistically significant among older women (*p* < 0.001) and older men (*p* = 0.038) but not among individuals younger than 57 years. Among older women, increasing social network trends were substantially more common among those with lower SEP compared with those with higher SEP (31.4% vs. 17.4%). In contrast, the majority of older men—regardless of SEP—remained in the constant high SNI category, with relatively few individuals experiencing increasing network trends (*n* = 149; 8%). Decreasing networks were somewhat more common among lower-SEP men (17.3%) compared with higher-SEP men (11.3%).

### Mediation analysis

Table 3 presents the proportion of the total effect mediated (PM) by each social network indicator in the relationship between socioeconomic position and healthcare utilization. For outpatient healthcare utilization, several social network characteristics demonstrated significant mediating effects. Loneliness mediated 3.1% of the SEP–outpatient utilization relationship before UHC implementation, increasing to 4.8% after UHC implementation. Similarly, the marital dyad mediated 3.9% of the relationship before UHC and 7.3% after UHC. Community social networks and social trust showed comparatively smaller mediation effects for outpatient utilization.

**Table 3:** Proportion of the total effect mediated by social network characteristics in the association between socioeconomic position and healthcare utilization before (2007/08) and after (2014/15) Universal Health Coverage implementation

Mediator	Outpatient services 2007/08% (95% CI)	Outpatient services 2014/15% (95% CI)	Change	Posyandu program 2007/08% (95% CI)	Posyandu program 2014/15% (95% CI)	Change
Loneliness	3.1 (2.3–4.5)	4.8 (3.2–9.1)	+ 1.7	0.4 (0.3–0.9)	5.2 (2.6–27.5)	+ 4.8
Marital dyad	3.9 (2.7–7.5)	7.3 (5.7–9.9)	+ 3.4	4.9 (3.2–9.8)	7.7 (0.7–14.3)	+ 2.8
Contact with children	2.1 (1.4–3.9)	3.1 (2.1–5.6)	+ 1.0	3.6 (2.4–7.3)	13.6 (− 7.4–0.6)	—
Community social networks	0.7 (0.4–1.5)	1.6 (1.2–2.4)	+ 0.9	5.1 (3.1–11.9)	21.7 (12.4–74.9)	+ 16.6
Social trust	0.4 (0.3–6.4)	1.3 (0.9–2.3)	+ 0.9	0.8 (0.5–1.7)	1.4 (0.6–6.8)	+ 0.6
Social network dynamics	—	4.2 (3.3–5.7)	—	—	10.2 (2.7–15.2)	—

For *posyandu* programme utilization, community-level social networks played a more substantial mediating role. Community social networks mediated 5.1% of the SEP–*posyandu* relationship before UHC implementation, increasing markedly to 21.7% after UHC implementation. Loneliness also showed moderate mediation effects for this outcome.

Overall, the proportion of the SEP–healthcare utilization relationship mediated by social networks increased across most indicators between the two survey waves, with increases ranging from 0.6% to 16.6%. Finally, social network dynamics mediated 4.2% of the association between SEP and outpatient healthcare utilization and 10.2% of the association between SEP and *posyandu* programme utilization.

Sensitivity analyses (Supplementary Table 1) indicated modest robustness of the principal indirect effects to unmeasured mediator–outcome confounding. The indirect association between SEP and *posyandu lansia* utilization through community social networks yielded mediational E-values of 1.30 in both IFLS-4 and IFLS-5 (1.23 and 1.25, respectively, for the confidence-limit closest to the null). The IFLS-4 indirect effect through loneliness on outpatient utilization was more sensitive to potential unmeasured confounding (E-value 1.11; confidence-limit E-value 1.07), while the remaining examined indirect effects had confidence intervals including the null.

## Discussion

The present study examined whether social networks act as intermediate factors in the relationship between socioeconomic position and healthcare utilization among older adults in Indonesia, while also providing insights into the dynamics of social networks over time. Using two nationally representative survey waves collected before and after the implementation of Universal Health Coverage, the findings highlight the complex interplay between socioeconomic conditions, social relationships, and healthcare utilization.

First, our results showed a clear increase in healthcare utilization following the introduction of Universal Health Coverage, both for outpatient services and participation in the *posyandu lansia* programme. These findings suggest that the expansion of financial protection through Indonesia’s national health insurance scheme may have reduced some barriers to healthcare access. Similar trends have been reported in previous studies examining healthcare utilization patterns in Indonesia following the implementation of UHC [31, 32, 60]. However, despite the overall increase in utilization, socioeconomic inequalities in healthcare use remained evident, particularly for outpatient services where individuals with higher SEP continued to show higher utilization levels.

Participation in the *posyandu lansia* programme increased over time but remained comparatively low overall, particularly among individuals with higher SEP. Community-based health promotion programmes such as *posyandu* represent an important component of Indonesia’s strategy to address the growing burden of chronic diseases among ageing populations. Previous research has highlighted that factors such as community engagement, local leadership, and government support play a key role in determining the effectiveness and uptake of such programmes [36, 37, 67]. Given the significant mediating role of community social networks observed in our study, community-based social interventions may represent a promising strategy to strengthen participation in preventive services among older adults.

Our findings also provide new insights into the social patterning and dynamics of social networks among older Indonesian adults. Individuals with higher SEP were more likely to maintain stable and consistently high levels of social connectedness over time, whereas individuals with lower SEP showed more dynamic network trajectories, including both increasing and decreasing social network patterns. These findings suggest that social networks among older adults are not static but evolve over time and may be shaped by broader socioeconomic conditions. Further stratified analyses indicated that sex/gender and age play an important role in shaping these dynamics, particularly among older women. Increasing social network trends were most commonly observed among older women with lower SEP, while older men - irrespective of socioeconomic position - were more likely to remain in stable, high-network configurations. These patterns may reflect differences in gendered life course trajectories, caregiving roles, and social participation patterns. Previous studies in high-income countries have similarly highlighted how the intersection of gender, age, and socioeconomic position shapes the structure and function of social networks in later life [40, 49, 68, 69].

At the same time, these findings may reflect structural constraints affecting ageing populations in low- and middle-income settings. Unlike in many high-income contexts, where changes in social networks are often interpreted as voluntary processes driven by socioemotional selectivity or other theories of “choice” [70], network changes in LMIC settings may be more strongly shaped by structural constraints such as economic vulnerability, health problems, or limited formal support systems. Similar interpretations have been proposed in studies of older adults in other LMIC contexts, including rural South Africa [13]. In such contexts, individuals may actively seek to expand their networks in order to mobilize support and cope with social or health-related challenges.

Assuming that the intersection of older age, female sex/gender, and low socioeconomic position represents a particularly vulnerable social position, the increasing network dynamics observed among older low-SEP women in our study may reflect attempts to mobilize support in response to unmet health and social needs. These dynamics may be related to life-course transitions such as widowhood, deteriorating health, or disability. However, because the observed “increase” in networks was derived from a summary social network index (SNI) combining several indicators, our findings cannot be interpreted as direct evidence of network growth or shrinkage. At the same time, several of the indicators included in the SNI—such as marital status, contact with children, and community participation—may reflect structural aspects of network size or diversity and thus potential sources of social support.

Our previous work has likewise identified older Indonesian women as a particularly vulnerable population group whose health is strongly shaped by the diversity of their social networks [15]. In addition, our qualitative research in rural Java suggests that older adults may actively renegotiate care relationships—sometimes against prevailing social norms—in order to “bargain a sense of security” and cope with loneliness [71]. Taken together, these findings suggest that increasing network dynamics among older low-SEP women may represent adaptive strategies to address unmet needs in contexts where formal support systems remain limited. In the context of ongoing social change, demographic ageing, and the growing “feminization of ageing”, this population group may increasingly require additional support beyond what informal family networks alone can provide.

The mediation analyses revealed that several social network characteristics contributed to explaining the association between SEP and healthcare utilization. In particular, loneliness and the marital dyad emerged as important mediators of outpatient healthcare utilization, while community-level social networks played a stronger mediating role in participation in the *posyandu lansia* programme. These findings highlight that both structural and functional aspects of social relationships may influence pathways into healthcare [72, 73]. The increasing mediating role of loneliness is particularly noteworthy. Loneliness has increasingly been recognized as a major public health concern, particularly among ageing populations [22, 71, 74–76]. Previous studies have shown that loneliness is associated with a wide range of adverse health outcomes and may also influence patterns of healthcare utilization [26–28]. Our findings suggest that loneliness may represent an important pathway linking socioeconomic disadvantage to healthcare use among older adults. However, the relationship between loneliness and healthcare utilization is complex and may partly reflect the fact that loneliness is closely intertwined with both physical and mental health condition.

Another important finding concerns the comparatively smaller mediating role of contact with adult children. While family networks are traditionally viewed as central sources of support in later life, our results suggest that children may play a less prominent role in facilitating healthcare utilization than spouses or community networks, particularly following the expansion of formal healthcare coverage. These findings may reflect broader transformations in intergenerational relationships occurring in many rapidly changing societies. Debates in social gerontology have long focused on the role of families in providing care for ageing populations and how this role interacts with formal welfare systems. Some scholars have argued that the expansion of welfare states may “crowd out” family-based support, while others suggest that formal and informal support systems may complement each other [77–79]. Our findings provide tentative support for the idea that informal care relationships remain important but may evolve alongside expanding formal health systems [80–84]. In particular, the growing mediating role of spousal relationships observed in our study is consistent with the notion of hierarchical caregiving arrangements in which spouses often act as primary caregivers in later life.

Overall, our findings highlight the importance of considering social relationships as part of the broader social determinants of healthcare utilization. In settings where ageing populations are increasing rapidly and health systems are undergoing major reforms, understanding how social networks interact with structural healthcare policies is crucial for designing effective public health strategies. These findings suggest that expanding formal healthcare coverage alone may not be sufficient to reduce socioeconomic inequalities in healthcare utilization among older adults. Policies aimed at improving access should also consider the social resources through which older people obtain information, practical assistance, encouragement, and connections to community-based services. Strengthening community-based programmes such as *posyandu lansia*, while identifying older adults with limited social support or weak community integration, may help ensure that improvements in healthcare coverage translate into more equitable service use. At the same time, interventions should avoid assuming that social networks are uniformly beneficial, as their availability and function are themselves socially patterned.

Several limitations should be considered when interpreting the findings of this study. First, although we applied a causal mediation framework, the analyses were based primarily on repeated cross-sectional data, which limits the ability to establish clear temporal ordering between exposure, mediator, and outcome variables. Reverse causality is therefore possible. For example, healthcare utilization may influence social network structures through increased reliance on family members or caregivers. In addition, healthcare utilization was measured over a relatively short recall period and without direct adjustment for healthcare need. Consequently, non-utilization cannot necessarily be interpreted as unmet need or restricted access, as it may also reflect good health, absence of healthcare needs, or lack of scheduled service activity during the observation period. Second, some of the measures used to capture social networks—such as loneliness—reflect subjective perceptions of social connectedness rather than structural network characteristics. While loneliness represents an important psychosocial dimension of social relationships, it may capture different aspects of social experiences than structural network measures. Third, although the IFLS provides rich population-based data, unmeasured factors such as cultural norms, health literacy, informal caregiving practices, and structural or geographic access to healthcare—including proximity to healthcare facilities—may also influence healthcare utilization patterns. Indonesia’s substantial ethnic and cultural heterogeneity was not directly accounted for at the individual level, although broader contextual variation was partly represented through the province-level social trust measure. In addition, migration between survey waves may have contributed to observed changes in social networks and could not be disentangled from other sources of network dynamics. Finally, our measure of social network dynamics was based on a summary index combining several network indicators, which limits the ability to disentangle the specific network processes underlying observed changes.

## Conclusions

This study provides new evidence on the role of social networks as intermediate factors in the relationship between socioeconomic position and healthcare utilization among older adults in Indonesia. Using nationally representative data collected before and after the implementation of Universal Health Coverage, the findings highlight the continued importance of social relationships in shaping access to healthcare in ageing populations. The results demonstrate that social networks contribute to explaining socioeconomic inequalities in healthcare utilization, while also revealing important gendered and socioeconomic differences in social network dynamics. These findings underscore the need to consider both formal health system reforms and informal social support structures when addressing healthcare access among ageing populations. In the context of Indonesia’s ongoing demographic and epidemiological transitions, policies aimed at strengthening community-based support structures, addressing loneliness among older adults, and integrating formal and informal care networks may play an important role in promoting equitable healthcare access.

## Supplementary Information

Below is the link to the electronic supplementary material.

Supplementary Material 1

## Data Availability

The datasets analysed during the current study are publicly available from the RAND Corporation Indonesian Family Life Survey (IFLS) data repository. The IFLS datasets can be accessed upon registration at: https:/www.rand.org/well-being/social-and-behavioral-policy/data/FLS/IFLS.html . No new datasets were generated during the current study.

## Abbreviations

HCU: Healthcare utilization
IFLS: Indonesian family life survey
LMICs: Low–and middle–income countries
NEM: Network epidsode model
SEP: Socioeconomic position
UHC: Universal health coverage
