# Supplementary material for: Socioeconomic inequalities in healthcare utilization among older adults in Indonesia: social network mediation and dynamics before and after Universal Health Coverage
Source: BMC Health Serv Res. 2026 Nov 11;26:1563. doi: 10.1186/s12913-026-16046-0 (PMC13756919; doi:10.1186/s12913-026-16046-0)
Supplement: Supplementary file 1 [file 12913_2026_16046_MOESM1_ESM.docx]

Supplementary Table 1: Sensitivity analyses of selected mediation models using mediational E-values in IFLS-4 and IFLS-5.

| **Wave** | **Mediator** | **Outcome** | **NIE RR** | **95% CI** | **E-value** | **E-value CI** |
| --- | --- | --- | --- | --- | --- | --- |
| IFLS-4 | Loneliness | Outpatient | 0.991 | 0.985–0.996 | 1.11 | 1.07 |
| IFLS-4 | Community networks | Posyandu | 1.057 | 1.035–1.077 | 1.30 | 1.23 |
| IFLS-4 | Loneliness | Posyandu | 1.002 | 0.985–1.020 | 1.05 | 1.00 |
| IFLS-5 | Loneliness | Outpatient | 0.997 | 0.991–1.002 | 1.06 | 1.00 |
| IFLS-5 | Community networks | Posyandu | 1.055 | 1.040–1.070 | 1.30 | 1.25 |
| IFLS-5 | Loneliness | Posyandu | 1.009 | 0.998–1.021 | 1.10 | 1.00 |

**Note.** NIE RR = natural indirect effect on the risk-ratio scale. Higher E-values indicate greater robustness to unmeasured mediator–outcome confounding. Analyses were adjusted for age and sex/gender; 95% confidence intervals were obtained using 1,000 bootstrap repetitions.
